# Staged external fixation and dorsal mini-plate fixation for a distal radius fracture with an overlying volar burn

**DOI:** 10.1051/sicotj/2026074

**Published:** 2026-10-07

**Authors:** Takahiro Yamazaki, Yusuke Matsuura, Seiji Ohtori, Takane Suzuki

**Affiliations:** Department of Orthopaedic Surgery, Graduate school of Medicine, Chiba University 1-8-1 Inohana Chuo-ku Chiba 260-8670 Japan

**Keywords:** Distal radius fracture, Burn, Collocated injury, External fixation, Dorsal plating

## Abstract

Burns combined with fractures are uncommon and become particularly challenging when the burn overlies the surgical corridor. We report a 51-year-old woman with an intra-articular distal radius fracture and an overlying deep volar ulnar wrist burn (deep partial-thickness to full-thickness) after jumping from a second floor during a house fire. A spanning external fixator was applied through unburned skin on the day of injury while the burn was managed conservatively by plastic surgeons. Ten days later, definitive fixation was performed through an intact dorsal approach with articular reduction, bone substitute filling, and fragment-specific fixation using two low-profile 2.0-mm dorsal mini-plates; the extensor retinaculum was interposed at closure to reduce tendon irritation. Although intraoperative stress testing after plating showed no gross instability, the spanning fixator was retained for 4 weeks because construct rigidity was a concern and volar splinting would interfere with burn dressing care. At 14 months after injury (3 months after plate removal), the patient had good wrist motion, 80.3% grip strength versus the contralateral side, PRWE 16.5, and QuickDASH 20.5, without infection or pin-tract problems. Staged stabilization that avoids burned skin and uses a dorsal safe corridor may be a feasible option in selected patients while facilitating ongoing burn management.

## Introduction

Combined burn and fracture injuries are rare but can cause substantial morbidity [1]. When the burn overlies the fracture site (a collocated injury), standard incisions and implants may traverse compromised skin, raising concern for infection, wound breakdown, and interference with burn care [1, 2]. Although early fracture stabilization in burn patients has been advocated, the strategy must be individualized according to burn depth, the need for excision/coverage, and the feasibility of avoiding the burned corridor [3, 4].

A targeted search of PubMed and Google Scholar (last accessed February 5, 2026) found no case report of a distal radius fracture with an overlying full-thickness volar wrist burn requiring avoidance of a volar approach and staged dorsal fixation.

## Case report

A 51-year-old woman sustained multiple injuries after jumping from the second floor during a house fire. On presentation, she had a left intra-articular distal radius fracture (AO/OTA 2R3C3), multiple vertebral fractures, and burns to the face and extremities (total body surface area approximately 4%). The wrist was markedly swollen from fracture displacement. There was no neurological deficit; the radial pulse was readily palpable, and there were no clinical findings suggestive of compartment syndrome. The burn measured approximately 13 × 8 cm and extended across the volar wrist and distal forearm. The volar wrist component was assessed as a deep partial-thickness burn and the ulnar component as a full-thickness burn by the plastic surgery team. The burn directly involved the usual volar plating corridor, whereas the dorsal wrist skin was largely intact (Figure 1). Radiographs and computed tomography demonstrated a markedly displaced and comminuted intra-articular distal radius fracture. The articular surface was divided into three major fragments. The ulnar articular component was further split into volar and dorsal fragments with additional small comminuted fragments. In particular, the lunate facet fragment was markedly displaced. On the day of injury, a wrist-spanning external fixator designed for distal radius fractures was applied. Two distal pins were inserted into the proximal second metacarpal and two proximal pins into the radius, proximal to the planned dorsal surgical field; all pins were placed through unburned skin. The two metacarpal pins were secured with one connector and the two radial pins with another, linked by a single bar. Satisfactory fracture length and alignment were obtained with longitudinal traction (Figure 2). Posterior spinal instrumentation was performed under the same anesthetic session. The plastic surgery team elected conservative burn management with serial wound care and dressings.

**Figure 1:**
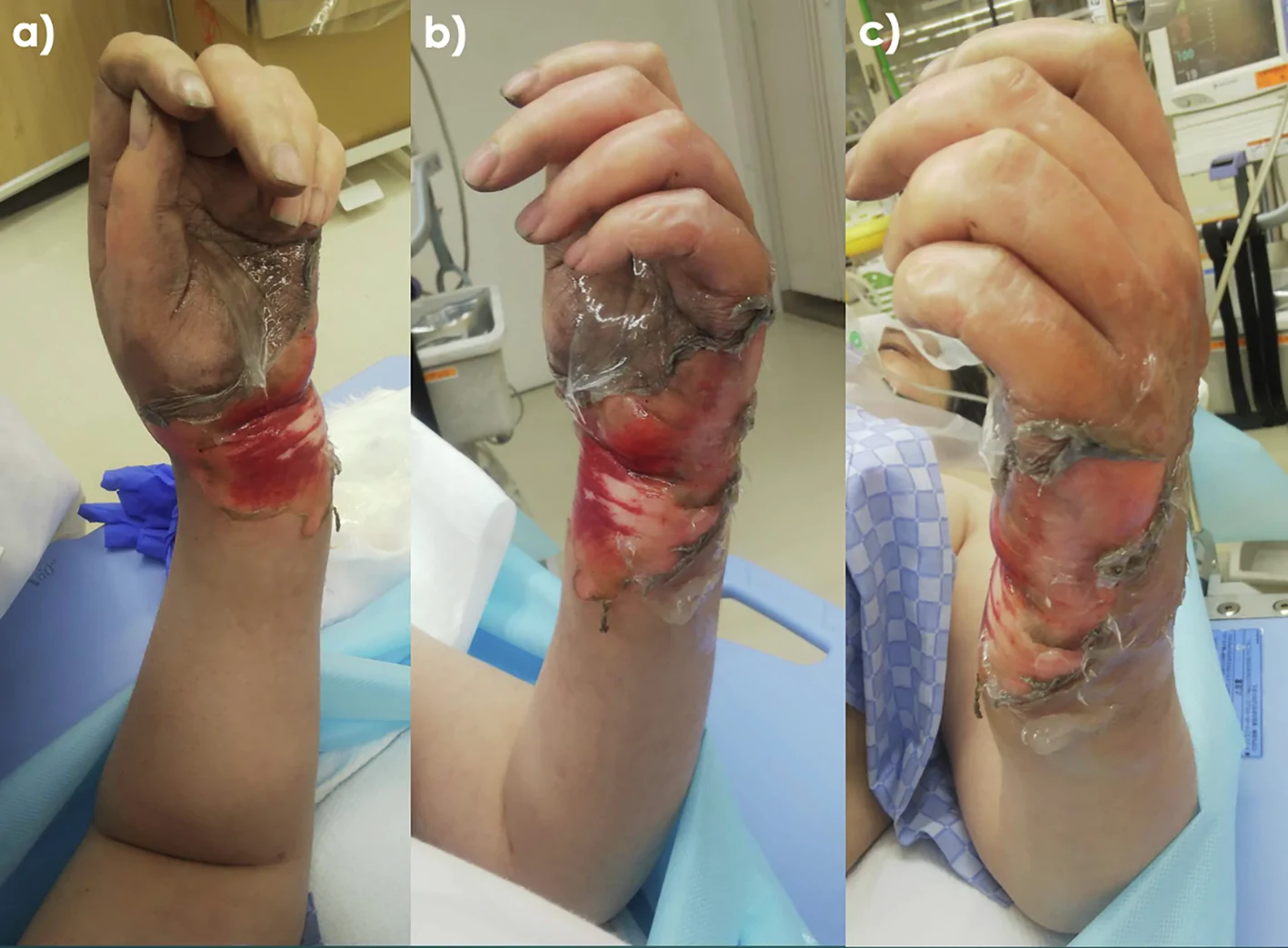
Clinical appearance of the wrist at initial presentation. Clinical photographs of the injured wrist on arrival showing an approximately 13 × 8-cm burn extending across the volar wrist and distal forearm. The volar wrist component was clinically assessed as a deep partial-thickness burn, whereas the ulnar component was assessed as a full-thickness burn by the plastic surgery team. The burn directly overlapped the standard volar plating corridor.

**Figure 2:**
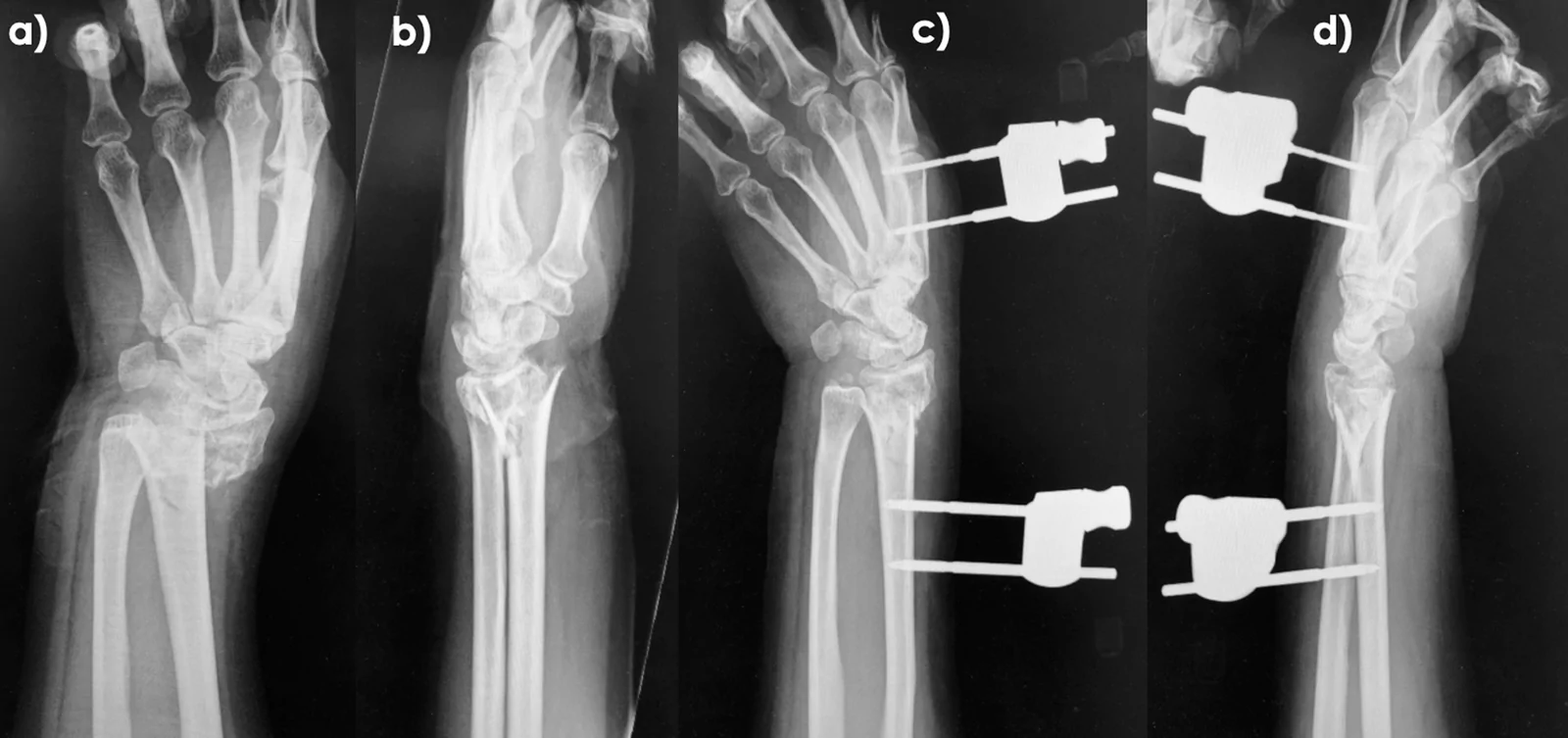
Radiographs before and after provisional spanning external fixation. (a, b) Initial anteroposterior and lateral radiographs demonstrating a markedly displaced and comminuted intra-articular distal radius fracture (AO/OTA 2R3C3). The articular surface was divided into three major fragments, with volar and dorsal splitting and additional comminution of the ulnar articular component. (c, d) Postoperative anteroposterior and lateral radiographs after spanning external fixation, showing restoration of good alignment.

Definitive fixation of the distal radius was performed 10 days after injury, after stabilization of the patient’s general condition and treatment of the concomitant spinal injuries. Fracture length and alignment were maintained with the spanning external fixator, allowing this short delay. Because the dorsal skin remained intact and provided a safe alternative surgical corridor, early burn excision and skin grafting or flap coverage solely to permit a volar approach was not pursued. External fixation alone was considered less suitable because direct reduction and stable fixation of the markedly displaced and comminuted intra-articular fragments were desirable. Because the volar burn remained unsuitable for incision, a dorsal approach through intact skin was used. A dorsal longitudinal incision approximately 5 cm in length was made, and the second through fourth extensor compartments were exposed. The extensor pollicis longus tendon was released from the third compartment. The articular fragments were directly reduced, with particular attention to the markedly displaced lunate facet fragment. After reduction and elevation of the lunate facet fragment, the associated subchondral bone void was filled with ReBOSSIS. Fragment-specific fixation was performed using two low-profile 2.0-mm mini-plates, one positioned beneath the second extensor compartment and the other beneath the fourth extensor compartment. Four screws were placed to support the articular surface. The extensor retinaculum was interposed between the plates and extensor tendons to minimize direct tendon-implant contact. Free gliding of the extensor tendons without interference from the plates was confirmed intraoperatively. The spanning frame was dismantled intraoperatively to facilitate reduction and internal fixation. After internal fixation, the external fixator was temporarily removed, and construct stability was assessed fluoroscopically during wrist flexion, extension, radial deviation, and ulnar deviation. No secondary displacement of the fracture fragments was observed. Nevertheless, the external fixator was reassembled for two reasons. First, it provided supplementary mechanical protection because the definitive construct consisted of two 2.0-mm mini-plates in a comminuted intra-articular fracture. Second, it avoided the need for a volar splint and preserved access to the volar burn for daily wound care (Figure 3).

**Figure 3:**
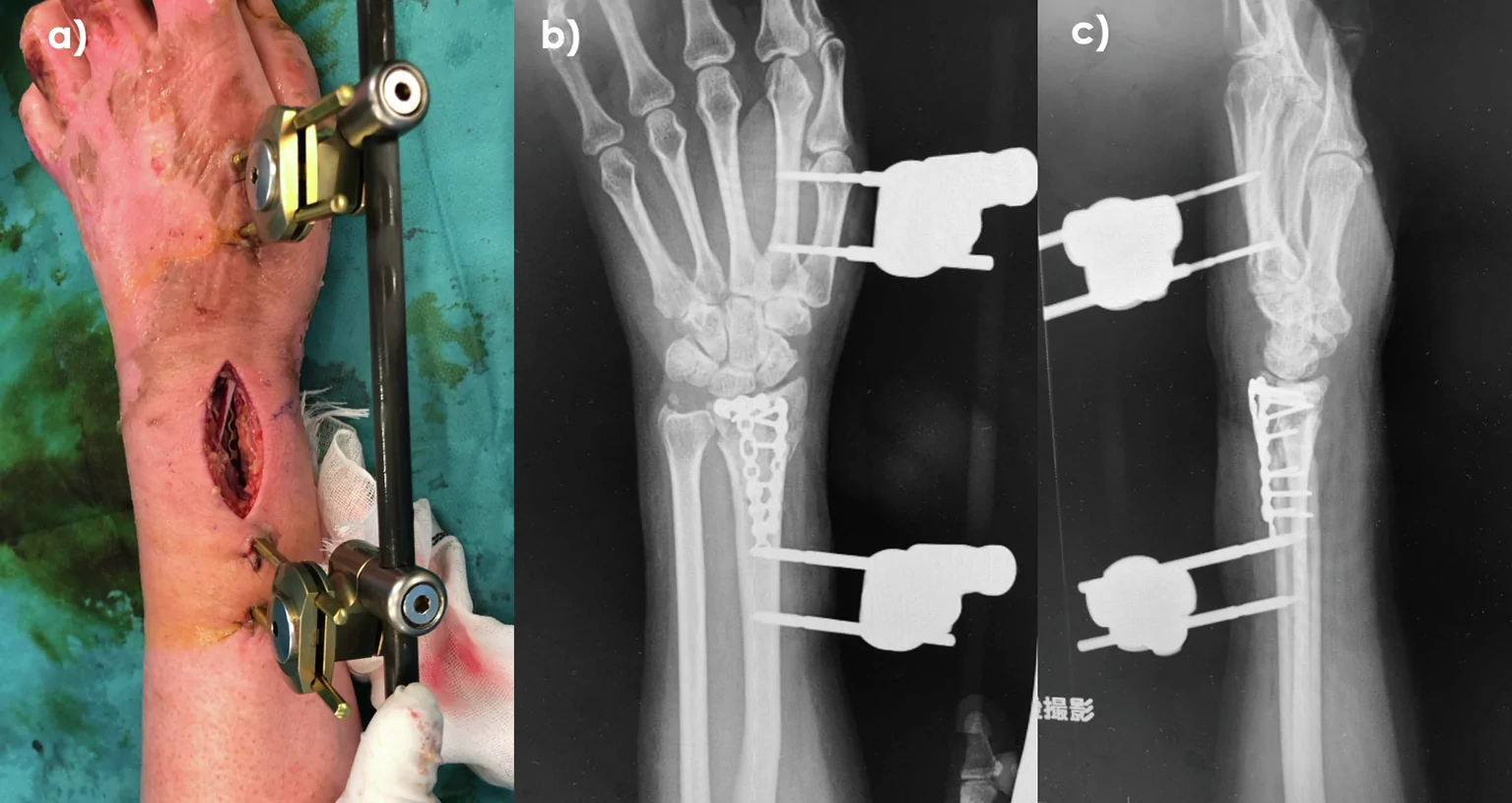
Findings at staged definitive surgery and postoperative alignment. (a) Intraoperative photograph demonstrating a dorsal approach through intact skin. Two low-profile 2.0-mm mini-plates were positioned beneath the second and fourth extensor compartments, respectively, and the extensor retinaculum was interposed between the plates and extensor tendons. (b, c) Post-fixation anteroposterior and lateral radiographs demonstrating maintained articular reduction and alignment. The spanning external fixator was retained for 4 weeks as supplementary mechanical protection while preserving access for daily burn care.

Active and passive finger range-of-motion exercises were encouraged from the early postoperative period, and forearm rotation was permitted while the spanning external fixator remained in place. The burn was assessed and dressed daily by the plastic surgery team. The external fixator was intended as temporary supplementary protection and was removed as planned at 4 weeks without predefined radiographic or functional criteria. Wrist motion was initiated after external-fixator removal and progressed as tolerated according to pain. Loading was initiated at approximately 5 kg at 1 month and gradually increased, with unrestricted loading from 3 months. There were no surgical site infections or pin tract problems. Radial inclination, volar tilt, and ulnar variance were 16.4°, 4.4°, and 1.1 mm immediately after definitive fixation and 16.8°, 6.7°, and 0.8 mm at final follow-up, respectively, demonstrating maintenance of reduction. Radiographs demonstrated fracture union. After union was confirmed, the dorsal mini-plates were electively removed at 10 months, primarily at the patient’s request, not because of extensor tendon irritation. No extensor tendon irritation or dysfunction was observed during follow-up. Final assessment was performed 3 months after plate removal (14 months after injury). The patient reported mild activity-related pain and had a hypertrophic scar at the volar wrist. Wrist range of motion was 60° flexion, 70° extension, 90° supination, and 80° pronation, with good finger motion (Figure 4). Grip strength was 22.5 kg on the injured side and 28.0 kg on the contralateral side (80.3%). Patient-reported outcomes were PRWE 16.5 and QuickDASH 20.5 (work module 25; sports/performing arts module not completed).

**Figure 4:**
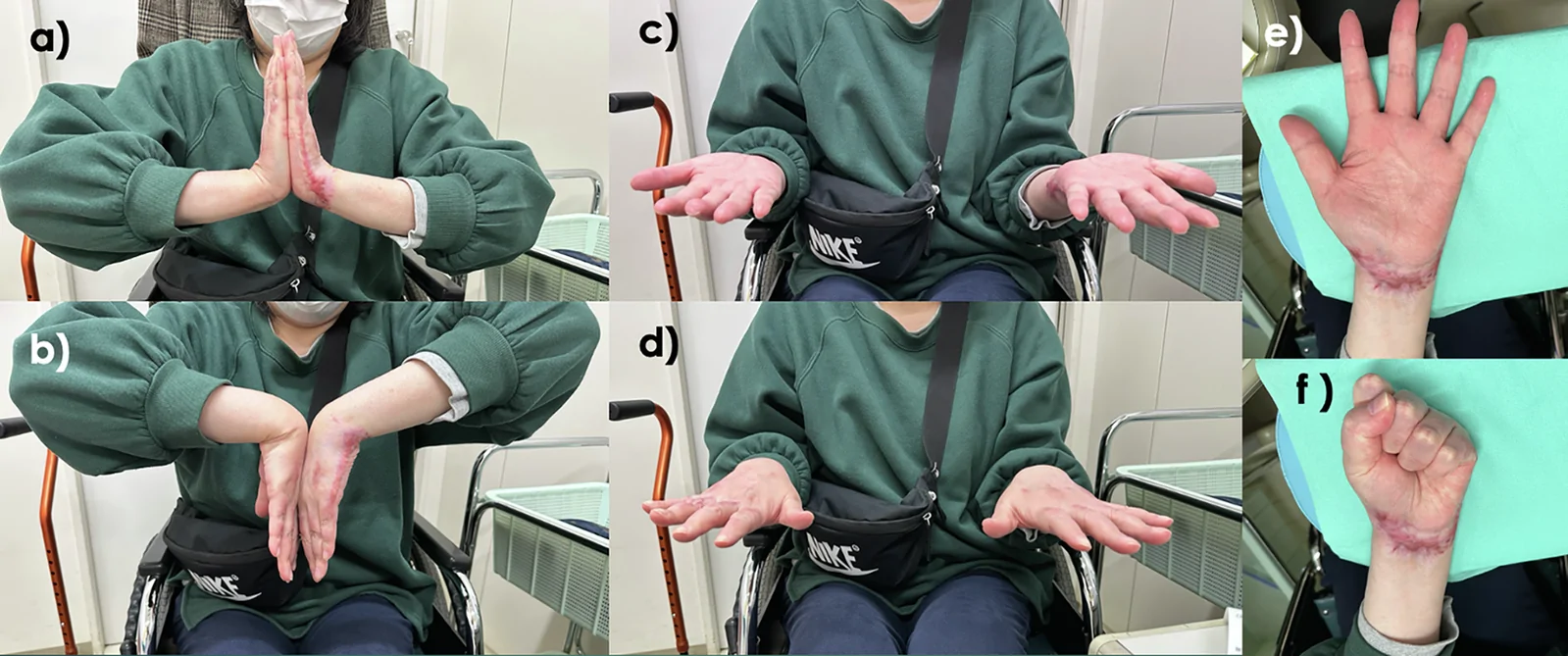
Final clinical appearance and range of motion. (a, b) Wrist flexion and extension at final follow-up. (c, d) Forearm pronation and supination at final follow-up. (e, f) Good finger motion at final follow-up.

## Discussion

This case highlights two recurring dilemmas in collocated burn–fracture injuries: (1) stabilizing the fracture without violating compromised skin and (2) maintaining immobilization without obstructing ongoing burn care. Contemporary reviews emphasize that timing and method of fixation should be individualized based on burn depth, contamination, physiologic status, and the feasibility of using a safe soft-tissue corridor [3, 4]. Observational series of collocated injuries suggest that complications relate strongly to soft-tissue status and overall injury burden, reinforcing the need to avoid incisions and pins through burned skin when feasible [1, 2].

In the present patient, the full-thickness component of the volar wrist burn directly overlaid the standard volar approach. Options included early excision/coverage to permit volar plating, delayed internal fixation after burn healing, or definitive external fixation. Because the fracture was markedly displaced and comminuted, with three major articular fragments and substantial displacement of the lunate facet fragment, direct articular reduction and stable internal fixation were preferred to external fixation alone. The plastic surgery team selected conservative burn management, and the intact dorsal skin provided a safe alternative surgical corridor. Thus, early burn excision and skin grafting or flap coverage were not pursued solely to facilitate volar plating. Once the patient’s general condition permitted definitive surgery, further delay was unnecessary. Minimally invasive volar plating has been reported for distal radius fractures with forearm skin problems [5], but even limited volar incisions would have crossed injured skin. We therefore chose staged spanning external fixation to restore length and alignment while allowing access to the burn wound, followed by definitive dorsal fixation through intact skin.

Another practical issue was postoperative immobilization. The rationale for retaining the spanning external fixator was twofold. Mechanically, it supplemented the two 2.0-mm mini-plates despite satisfactory fluoroscopic stress testing. In practice, it avoided the need for a volar splint and maintained unrestricted access to the burn for daily wound care. The fixator was removed at 4 weeks without loss of reduction or pin-related complications.

This report is limited by its single-case design and favorable clinical course and therefore cannot establish a treatment algorithm. The described staged strategy should be regarded as a feasible option for selected patients in whom a severe volar burn compromises the standard surgical corridor. Potential disadvantages include wrist stiffness associated with spanning fixation, extensor tendon irritation from dorsal implants, pin-tract infection, the need for implant removal, and the possibility that subsequent burn excision or soft-tissue coverage may still be required.

## Conclusion

In selected patients with a distal radius fracture and a severe overlying volar burn, staged external fixation followed by dorsal mini-plate fixation through intact skin may be a feasible treatment option that preserves access for burn care. The indications and potential disadvantages of this strategy should be considered on a case-by-case basis.

## Data Availability

All data generated or analyzed during this study are included in this published article. Additional details are available from the corresponding author upon reasonable request.
